# A 3-D Rat Brain Model for Blast-Wave Exposure: Effects of Brain Vasculature and Material Properties

**DOI:** 10.1007/s10439-019-02277-2

**Published:** 2019-05-03

**Authors:** Ginu Unnikrishnan, Haojie Mao, Aravind Sundaramurthy, E. David Bell, Stewart Yeoh, Kenneth Monson, Jaques Reifman

**Affiliations:** 1 Department of Defense Biotechnology High Performance Computing Software Applications Institute, Telemedicine and Advanced Technology Research Center, United States Army Medical Research and Materiel Command, MCMR-TT, 504 Scott Street, Fort Detrick, MD 21702 USA; 2 The Henry M. Jackson Foundation for the Advancement of Military Medicine, Inc (HJF), 6720A Rockledge Drive, Bethesda, MD 20817 USA; 3 Department of Bioengineering, The University of Utah, 36 S. Wasatch Drive, Salt Lake City, UT 84112 USA; 4 Department of Mechanical Engineering, The University of Utah, 1495 E 100 S (1550 MEK), Salt Lake City, UT 84112 USA

**Keywords:** Rat cerebral vasculature, High-strain-rate material properties, Shock tube, Blast overpressure

## Abstract

Exposure to blast waves is suspected to cause primary traumatic brain injury. However, existing finite-element (FE) models of the rat head lack the necessary fidelity to characterize the biomechanical responses in the brain due to blast exposure. They neglect to represent the cerebral vasculature, which increases brain stiffness, and lack the appropriate brain material properties characteristic of high strain rates observed in blast exposures. To address these limitations, we developed a high-fidelity three-dimensional FE model of a rat head. We explicitly represented the rat’s cerebral vasculature and used high-strain-rate material properties of the rat brain. For a range of blast overpressures (100 to 230 kPa) the brain-pressure predictions matched experimental results and largely overlapped with and tracked the incident pressure–time profile. Incorporating the vasculature decreased the average peak strain in the cerebrum, cerebellum, and brainstem by 17, 33, and 18%, respectively. When compared with our model based on rat-brain properties, the use of human-brain properties in the FE model led to a three-fold reduction in the strain predictions. For simulations of blast exposure in rats, our findings suggest that representing cerebral vasculature and species-specific brain properties has a considerable influence in the resulting brain strain but not the pressure predictions.

**Electronic supplementary material:**

The online version of this article (10.1007/s10439-019-02277-2) contains supplementary material, which is available to authorized users.

## Introduction

Exposure to explosive devices is the leading cause of traumatic brain injury (TBI) in U.S. Soldiers deployed to Iraq and Afghanistan.^23^ Multiple studies provide evidence that such blast-induced TBI is caused primarily by penetrating and blunt trauma.^2^,^18^ In contrast, other studies postulate that the mere exposure to a blast wave can also cause brain injury, the so-called non-impact primary TBI.^6^,^21^ In the absence of human-exposure data, these studies invariably entail the use of animal models, rats in particular, with blast exposure induced by a shock tube.^3^,^17^ In this context, laboratory experiments allow us to detect and quantify potential injuries in the rat brain and computational finite-element (FE) models allow us to characterize the biomechanical responses of the brain and the possible mechanisms of injury.^17^,^19^,^25^ However, current FE models for blast-induced TBI in rats lack the necessary fidelity,^19^,^25^ as they do not consider the influence of cerebral vasculature and lack high-strain-rate material properties characteristic of blast exposures of the rat brain.

A handful of studies have investigated the contribution of the cerebral vasculature to the biomechanical response of the brain to mechanical loads.^9^,^10^,^24^ Using a two-dimensional FE model of the human head, Zhang *et al*. showed that the vasculature plays a major role in the biomechanical response (measured in terms of maximum principal strain and shear strain) of the human brain during head acceleration.^24^ Based on their findings, the authors suggested that cerebral vasculature should be explicitly represented in human-head models. Ho *et al*., using a three-dimensional (3-D) FE model of a human head, reported that inclusion of a cerebral vasculature system with non-linear material properties reduced the average peak strain by 5% when the head was exposed to a translational acceleration.^9^ Similarly, during exposure to blast loading, cerebral vasculature was reported to influence the predictions of maximum principal strain in the brain of a surrogate head model.^10^ Collectively, these studies suggest that cerebral vasculature is a contributor to the biomechanical behavior of brain tissues in response to external loading conditions.

In the absence of data on rat-brain tissues at high strain rates, previous FE blast-exposure models of the rat head incorporated either low-strain-rate material properties of the rat brain or high-strain-rate properties of the human head.^19^,^25^ These approximations may introduce errors in the FE predictions, as experimental studies have shown that brain material properties depend on species, region, and the applied strain rate.^12^ Recently, Haslach *et al*. performed high-strain-rate shear testing of brain tissues from adult, male Sprague–Dawley rats.^8^ Test results, obtained for the cerebrum, cerebellum, and brainstem regions, showed region- and rate-dependency of the brain tissues. Similarly, Bell *et al*. performed high-strain-rate tensile testing of the middle cerebral arteries from male Sprague–Dawley rats.^1^ The study, which was performed at strain rates ranging from quasi-static to 1000 s^−1^, showed rate-dependency in the material properties of the cerebral vasculature at strain rates beyond 700 s^−1^. With the availability of vasculature and material-testing data from rat-brain tissues, it is now possible to include species-specific brain material properties in the FE model and, thereby, address a major limitation of existing blast-exposure models of the rat head.

In this study, we developed a 3-D FE model of a rat head for blast-exposure simulations. In the FE model, we included the geometry of the cerebral vasculature and high-strain-rate material properties of rat-brain tissues and vasculature. Using this model, we quantified the biomechanical responses of the brain due to blast-wave exposure in a shock tube. We hypothesized that inclusion of the cerebral vasculature and species-specific high-strain-rate material properties of the brain in the FE model would influence the biomechanical response of the brain to blast waves.

## Materials and Methods

### Geometry and Finite Element Mesh of the Rat Head

**Figure 1:**
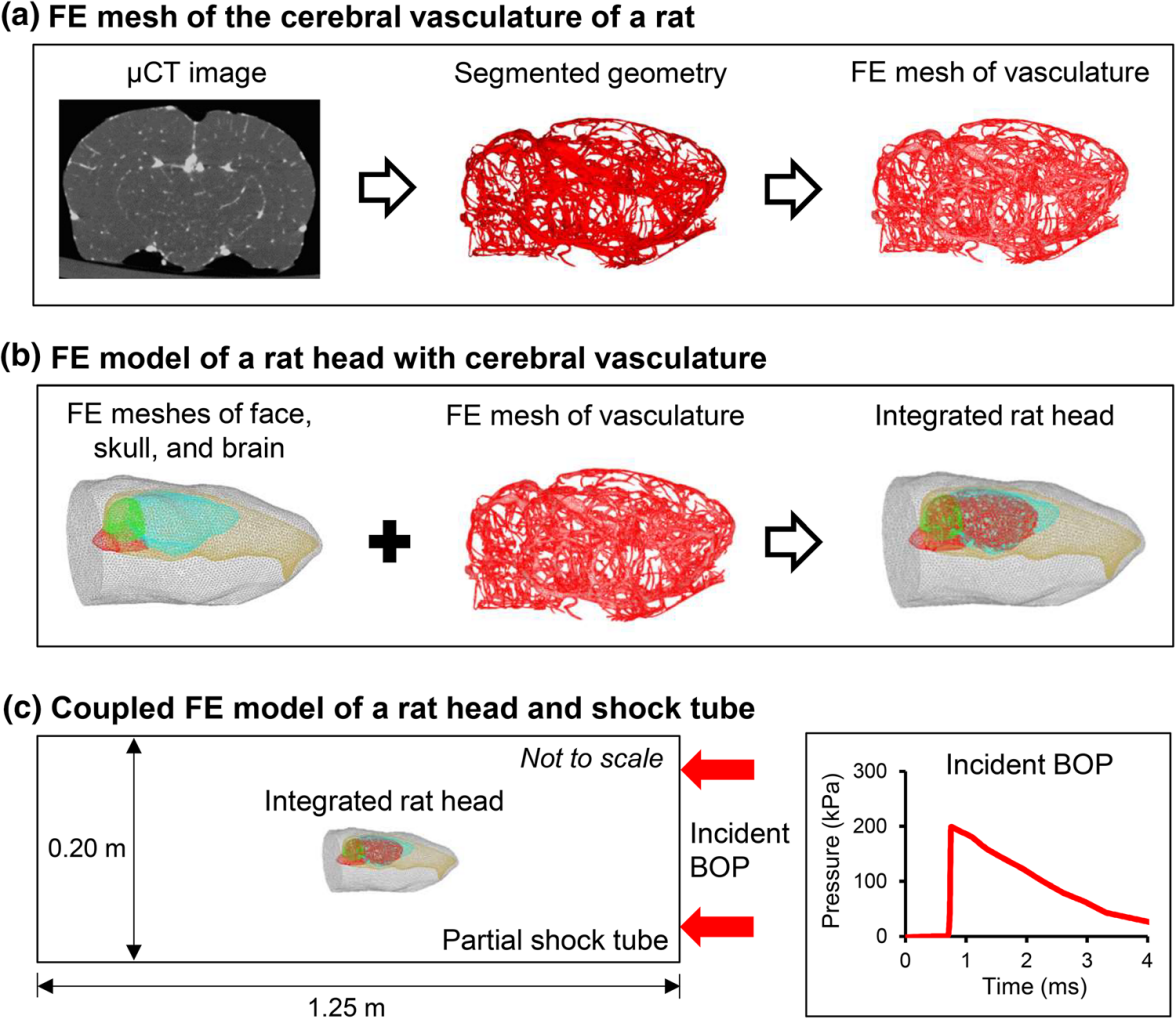
Rat head model with cerebral vasculature and coupled shock-tube model. (a) We developed a three-dimensional (3-D) finite element (FE) mesh of the cerebral vasculature of a male Sprague–Dawley rat from micro-computed (µCT) tomography images. (b) We integrated the FE meshes of the face, skull, facial bones, and brain with the FE mesh of the cerebral vasculature to develop a FE model of a rat head. (c) To perform the blast simulations, we coupled the integrated rat-head FE model with a 3-D partial shock-tube FE model. We performed blast simulations for incident blast overpressures (BOPs) ranging from 100 to 230 kPa with the rat head in a prone position and facing the blast wave (i.e., frontal orientation).

We obtained the geometry and created a FE mesh of the cerebral vasculature of a rat in three steps (Fig. 1a). First, following anesthetization of an adult, male Sprague–Dawley rat with isoflurane and its exsanguination *via* cardiac perfusion, we injected a high-contrast setting barium/iodine-based contrast agent (BriteVu, Scarlet Imaging LLC, Murray, UT) at a rate of 25 ml/min and allowed the agent to set for 30 min. Then, we decapitated the rat, removed the skin and musculature of the head, and fixed the rat head in 4% paraformaldehyde for 72 h. Finally, we extracted the brain from the skull and obtained micro-computed tomography (µCT; Siemens Inveon PET/CT, Siemens Medical Solutions USA, Inc., Malvern, PA) images of the brain with the contrast agent at a uniform resolution of 35 *μ*m per voxel. During scanning, the brain was immersed in a buffered saline solution. This procedure to obtain the geometry of the cerebral vasculature was approved by the Animal Care and Use Review Office of the U.S. Army Medical Research and Materiel Command, Ft. Detrick, MD, and the Institutional Animal Use and Care Committee at the University of Utah.

**Figure 2:**
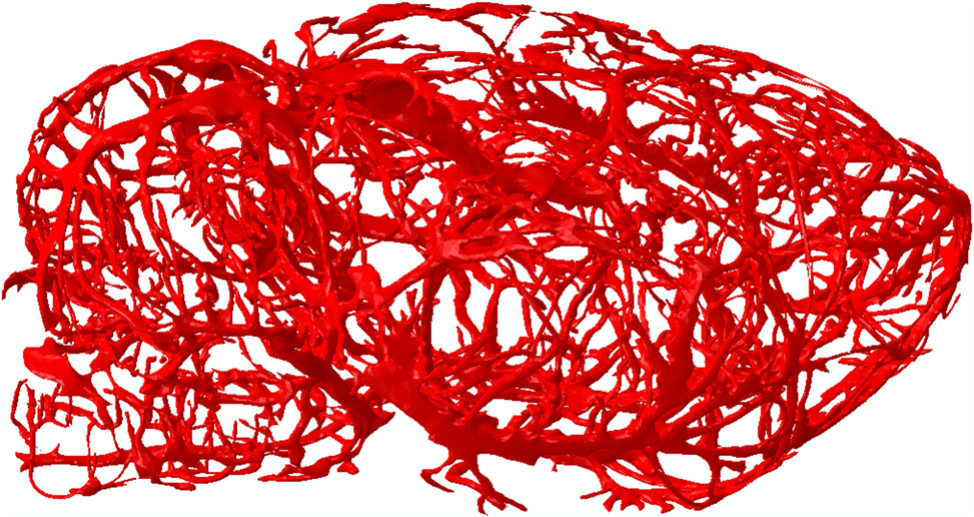
Three-dimensional geometry of the cerebral vasculature of a male Sprague–Dawley rat developed from micro-computed tomography images.

Second, we imported the µCT images into 3-Matic (Materialise, Leuven, Belgium) and segmented them using a semi-automated approach to create an initial geometry of the cerebral vasculature, including vessels down to the level of the penetrating arteries. Then, we manually improved the initial geometry by removing discontinuities as well as smoothing sharp angles (Fig. 2).

Finally, we imported the improved geometry of the cerebral vasculature into Hypermesh 2017.1 (Altair Engineering, Troy, MI) and meshed the geometry, using linear triangular shell elements of type S3. We modeled the cerebral vasculature as hollow pipes and did not consider any fluid flow through the vasculature. The cerebral vasculature was meshed with shell elements having an average minimum edge length of 0.07 mm. We assigned a constant shell thickness of 0.1 mm for the vasculature. Next, we imported the FE mesh into ABAQUS and performed a mesh-quality test. The elements had an average aspect ratio of 1.98, and only 1.3% had a ratio greater than 5.00. In addition, the average minimum and maximum angles were 33 and 93 degrees, respectively, and the average shape factor was 0.64.

We obtained the anatomical model of a rat, without the cerebral vasculature, from Duke University.^11^ Previously, we used this model of the rat anatomy to develop a computational model for performing whole-body thermal heat-stress analysis.^15^,^16^ After we extracted the geometry of the face (including the scalp and facial musculature), skull (including the facial bones), and brain from the anatomical model, we imported the geometry into ABAQUS v6.17 (Dassault Systèmes, Vélizy-Villacoublay, France). Next, we meshed the rat-head geometry with 250,935 quadratic (10-noded), tetrahedral elements (C3D10M) having an average minimum edge length of 0.7 mm (Fig. 1b). The edge length was determined from a mesh-convergence study that used the same boundary condition as that implemented in this study. Then, we checked the quality of the FE mesh in ABAQUS. The elements had an average aspect ratio of 1.66, with only 0.02% having a ratio greater than 5.00. The average minimum and maximum angles were 38 and 89 degrees, respectively, and the average shape factor was 0.65. Finally, from the FE mesh, we identified three major regions of the brain: the cerebrum, cerebellum, and brainstem.

We coupled the cerebral vasculature and the rat head FE meshes to develop an integrated model of a *rat head with cerebral vasculature* (RHwCV). To couple the models, first we determined the µCT image corresponding to the mid-transverse plane of the rat brain. Next, we determined the dimensions of the rat brain in the anterior–posterior and medial–lateral directions from this µCT image. Second, we scaled the 3-D rat head geometry such that the mid-plane of the rat brain closely matched the dimensions of the rat brain determined from the mid-transverse µCT image. After scaling, we evaluated the contours of the rat brain and cerebral vasculature and performed a rigid-body transformation to fit the cerebral vasculature within the rat brain. Finally, using embedding techniques in ABAQUS, we coupled the cerebral vasculature and the rat head FE meshes. In the RHwCV model, we treated the brain as the host elements and the cerebral vasculature as the embedded elements. We did not fill the interior of the cerebral vasculature (i.e., the lumen) with blood or any other material. However, although we did not explicitly model any material in the lumen, the embedded element technique used here assumes those regions to be filled with the host elements (i.e., brain elements). We assumed a no-slip boundary condition between the vasculature and brain elements, wherein the translation degrees of freedom of the brain and vasculature elements were constrained in all directions. However, the rotational degrees of freedom of the vasculature elements were not constrained by the brain elements.

### Material Properties of the Rat Head

Various material constitutive models and properties have been used to represent the behavior of the different components of an animal head for blast simulations.^4^,^10^,^14^,^19^,^22^,^25^,^26^ In this study, we considered the face as an incompressible material with an instantaneous elastic modulus of 7.5 MPa. We represented the deviatoric response of the face, using a one-term Ogden constitutive model with a one-term prony-series approximation. We modeled the skull as a compressible, linear-elastic material with an elastic modulus of 1 GPa and a Poisson’s ratio of 0.33 (Table 1).

**Table 1:** Material properties of the different components of the rat head.

Components	Density (kg m^−3^)	Elastic constants		Hyperelastic constants			Viscous constants	
		Elastic modulus (GPa)	Poisson´s ratio	Bulk modulus (GPa)	Shear modulus (kPa)	α	Relaxation modulus ratio	Decay constant (s^−1^)
Skull	1700	1.0	0.33					
Face	1100			2.0	2500.0	3.0	0.150	400
Cerebrum	1040			2.0	11.9	6.5	0.103	990
Cerebellum	1040			2.0	8.3	8.2	0.274	402
Brainstem	1040			2.0	12.3	4.7	0.112	1081
Cerebral vasculature	1040			2.0	525.0	7.5		

α material constant

We considered the brain tissue (i.e., the cerebrum, cerebellum, and brainstem) and the cerebral vasculature as incompressible materials. We used a one-term Ogden model with a one-term prony series representation to capture the deviatoric response of the brain. The material constants (Table 1) for the tissues of different brain regions were based on a recent experimental study on rat-brain tissues,^8^ in which shear tests at high-strain-rates performed on the cerebral, cerebellar, and brainstem tissues of male Sprague–Dawley rats. We derived the constants by calibrating the material model at strain rates of 33 and 333 s^−1^ for the cerebrum, 25 and 250 s^−1^ for the cerebellum, and 20 and 120 s^−1^ for the brainstem (Supplementary Fig. 1). We used a one-term Ogden model to capture the deviatoric response of the cerebral vasculature. Similar to those of brain tissues, the material properties of the cerebral vasculature were based on high-strain-rate axial tensile testing of the middle cerebral arteries of male Sprague–Dawley rats up to a maximum strain rate of 500 s^−1^ (Supplementary Fig. 2).^1^

### FE Model of the Shock Tube

We developed a 3-D FE model of a partial shock tube, similar to previous models,^13^,^19^,^20^ with a square cross-section of 0.20 m in width and 1.25 m in length (Fig. 1c). We modeled the air in the shock tube as an ideal gas (density of 1.23 kg m^−3^ and specific gas constant of 287 J kg^−1^ K^−1^) at a temperature of 300 K and meshed the air using Eulerian elements (EC3D8R). We placed the rat-head model in the middle of the partial shock tube at a distance of 0.08 m from the inlet surface, and coupled the two models using the coupled Eulerian–Lagrangian approach in ABAQUS. Using a biased-meshing technique, we assigned finer elements (edge length of 2.75 mm) for the air near the rat head and coarser elements (edge length of 7.50 mm) elsewhere. We selected the size of the elements through a mesh convergence study with the same boundary conditions as those considered in this study. The air in the shock tube was meshed with 773,175 elements.

### Blast Simulation

We determined the biomechanical responses of the rat brain, such as the intracranial pressure, shear stress, and maximum principal strain, for an incident static blast overpressure (BOP) of 200 kPa. We also determined the peak brain pressure for BOPs ranging from 100 to 230 kPa. The incident BOP-time profile was provided as a pressure boundary condition at the inlet of the shock tube. No boundary conditions were prescribed at the outlet of the shock tube. Along the sides of the shock tube, the velocity of the air perpendicular to the walls was constrained to zero. In addition, the nodes at the back of the rat head were constrained to zero displacement in all directions. We performed the simulations with the animal in a prone position, with the blast wave impact occurring in the frontal orientation.

To determine the contribution of the cerebral vasculature on the biomechanical responses, we compared the responses obtained with the RHwCV model to a FE model of the *rat head without the cerebral vasculature* (RHw/oCV). In addition, to assess the effect of material properties at high strain rates characteristic of BOP exposure, we developed a model, henceforth termed the *legacy* model, representative of previous FE approaches for simulating blast exposure of a rat head.^19^,^25^ In the legacy model, we did not include the cerebral vasculature and used high-strain-rate material properties of human brain tissues (instantaneous elastic modulus of 123 kPa, bulk modulus of 2.0 GPa, shear relaxation modulus ratio of 0.19, and decay constant of 700 s^−1^), as previously described.^19^ By comparing the responses predicted by the RHw/oCV and legacy models, we quantified the influence of different (rat vs. human) high-strain-rate material properties on the biomechanical responses to blast exposure in the rat brain. We also quantified the influence of vasculature thickness on the FE predictions by assigning a thickness of 0.010, 0.025, and 0.050 mm for the vasculature elements, in addition to the selected nominal value of 0.100 mm.

## Results

### Biomechanical Responses of the Rat Head

**Figure 3:**
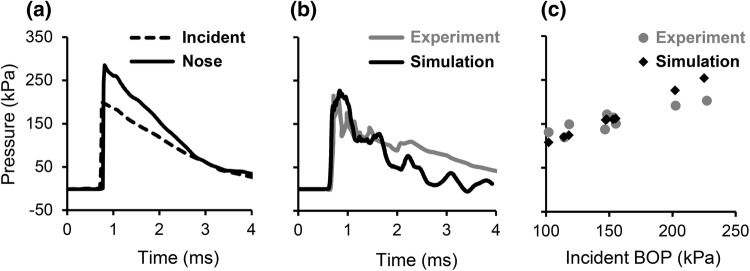
Comparison of pressures predicted by the finite element model of the rat head with cerebral vasculature (RHwCV) with those obtained from experimental studies. (a) Pressure–time profiles of the incident pressure and pressure at the nose of the rat. Comparison of experimental^19^ and RHwCV model-predicted (b) brain pressure–time profiles and (c) peak brain pressures. *BOP* blast overpressure.

From the simulations of the RHwCV model, we determined the biomechanical responses of the rat head (e.g., pressure, von Mises stress, maximum principal strain) when exposed to BOPs in a shock tube. For an incident BOP of 200 kPa, the surface pressure at the nose was nearly 1.5 times greater than the incident pressure, possibly due to blast-wave amplification (Fig. 3a). The pressure–time profile of the brain followed a trend similar to that of the incident BOP. We performed mesh convergence studies on the shock tube and the rat head at an incident BOP of 200 kPa. For a mesh size that was double that of the current model for the shock tube, pressure at the center of the brain changed by 2.6%. We performed mesh refinement studies on the rat head using four different meshes of the rat brain (Table 2). We observed that with an increase in the number of elements, the peak pressure at the center of the brain increased. The difference between the peak pressures predicted by the current model with 132,335 elements of the brain was only 1% different from the peak pressure predicted by a FE model with 76,090 brain elements. However, when compared to a FE model with 15,196 elements for the brain, the difference was 5%. In contrast to the change in the pressure, the peak strain did not change consistently with mesh size. To validate our model, we compared the predicted pressure–time profile with the experimentally reported profile,^19^ and observed a reasonable match (Fig. 3b). In addition, we compared the peak brain pressures (Fig. 3c) for BOPs ranging from 100 to 230 kPa with those reported in the literature.^19^ The percentage difference between the predicted and measured peak brain pressures ranged from 2 to 20%, with the greatest difference observed at a BOP of 230 kPa.

**Table 2:** Summary of mesh convergence performed on the rat head.

Model	Mesh size	Number of elements	Peak pressure (kPa)
1	0.10	15,196	215
2	0.08	29,502	220
3	0.06	76,090	225
4 (selected)	0.05	132,335	227

**Figure 4:**
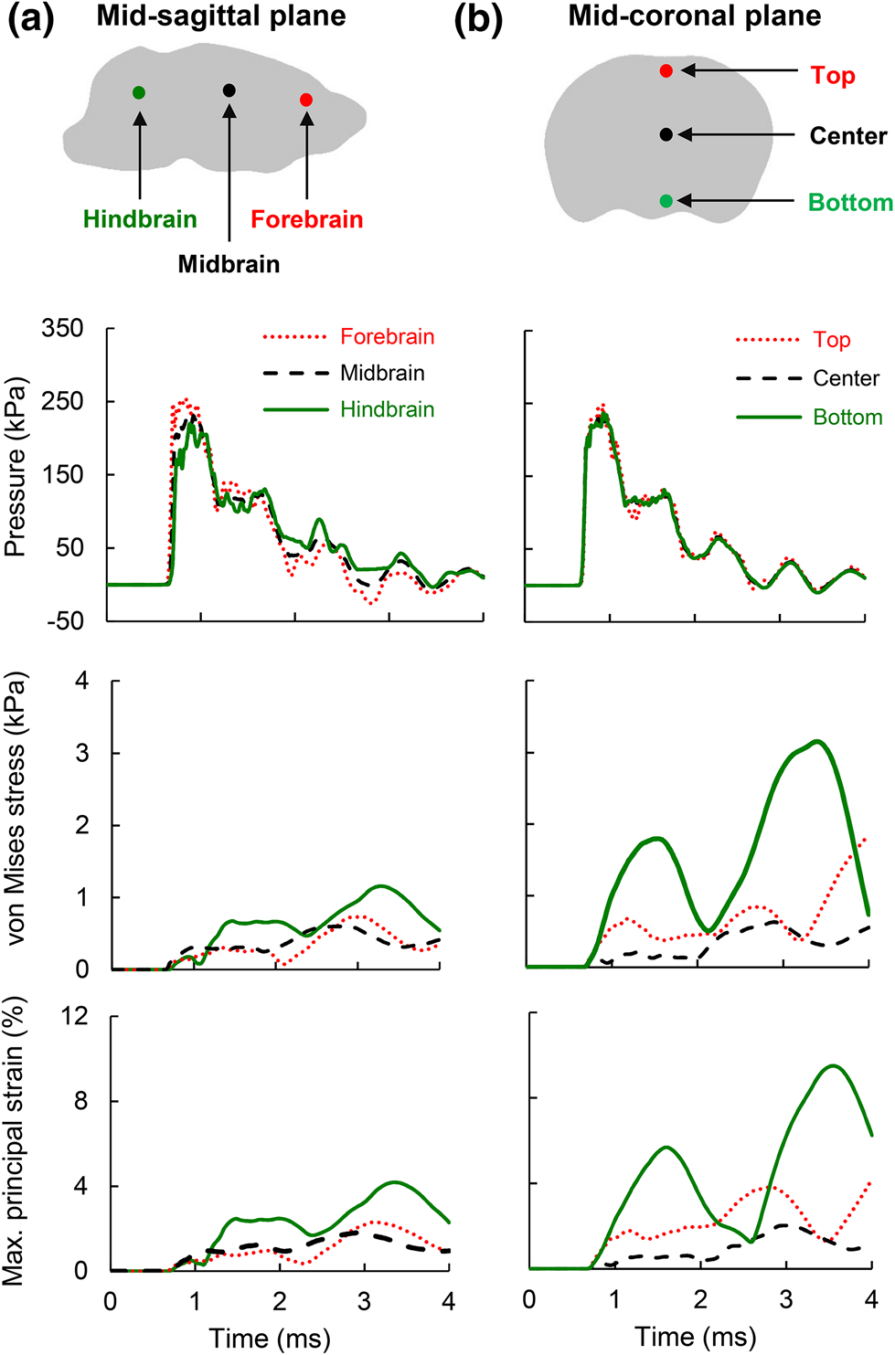
Temporal evolution of pressure, von Mises stress, and maximum principal strain predicted by the finite element model of the rat head with cerebral vasculature. The predictions are at different locations along the (a) mid-sagittal and (b) mid-coronal planes.

To understand the spatial variation in brain pressure, we determined the pressure at three locations along the mid-sagittal plane of the brain corresponding to the forebrain, midbrain, and hindbrain (Fig. 4a). Peak brain pressures at the midbrain and hindbrain were, respectively, 9 and 14% lower than the peak pressure at the forebrain. In addition, we determined pressures at the top, center, and bottom regions along the mid-coronal plane of the brain (Fig. 4b). The peak brain pressure at the center was only 8% and 3% lower than the pressures at the top and bottom, respectively. The magnitude of the shear stress (i.e., the von Mises stress) in the brain was two orders of magnitude smaller than the intracranial pressure. The von Mises stress developed ~ 2.5 ms later in the simulation, and showed considerable oscillations in the mid-sagittal (Fig. 4a) and mid-coronal planes (Fig. 4b).

**Figure 5:**
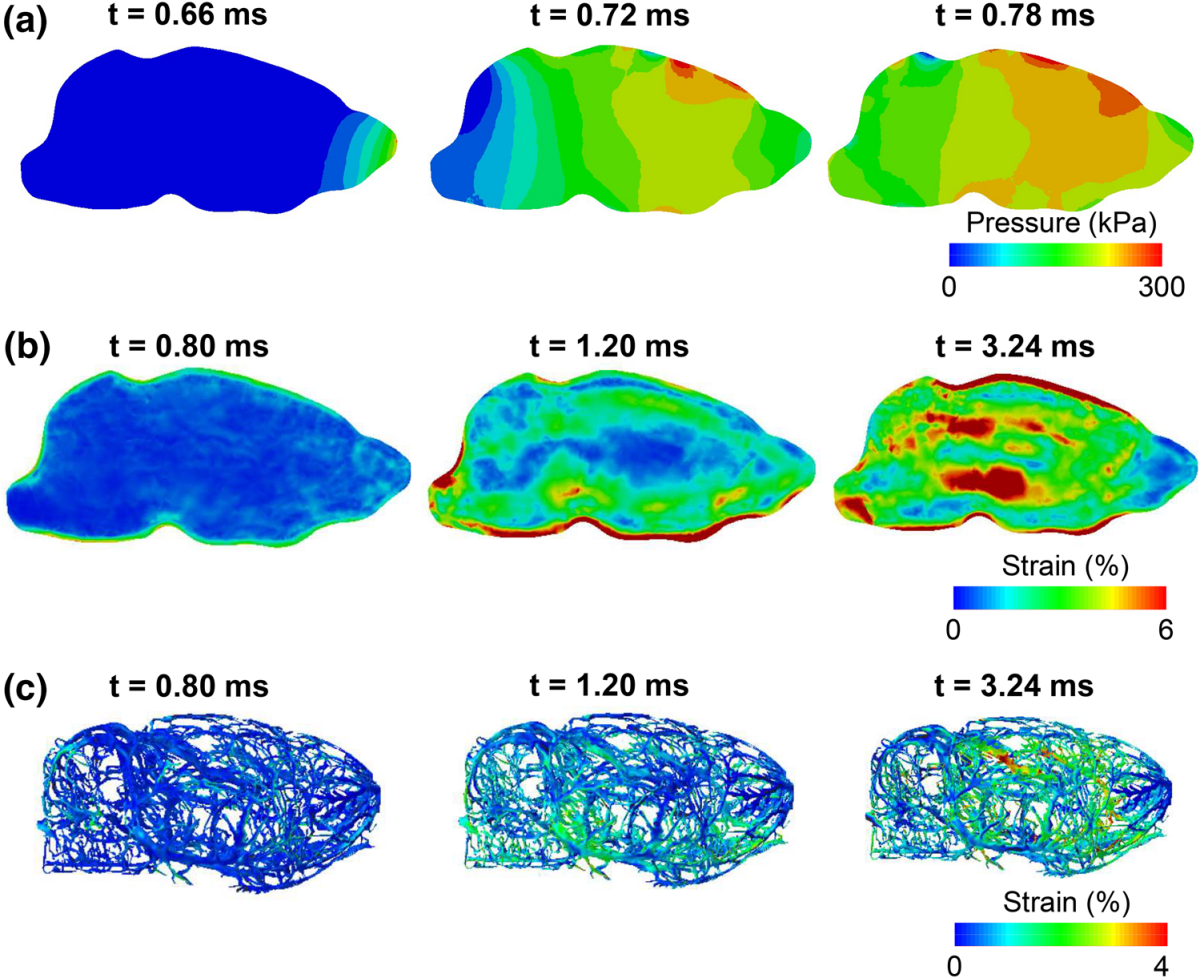
Temporal evolution of biomechanical responses predicted by the finite element model. Predictions are for (a) brain pressure at the mid-sagittal plane, (b) brain strain at the mid-sagittal plane, and (c) strain in the cerebral vasculature.

The maximum principal strain–time profile, similar to the von Mises profile, showed considerable oscillations at all locations in the mid-sagittal and mid-coronal planes (Fig. 4, bottom panel). The peak strain at the bottom of the mid-coronal plane was nearly four times greater than the strain at the center. The maximum principal strain in the brain and cerebral vasculature started in the peripheral regions of the brain and propagated towards the center of the brain with time (Figs. 5b and 5c). In contrast, the pressure wave traveled from the front (the right) to the back (the left) of the brain along the direction of blast-wave propagation (Fig. 5a). The strain in the cerebral vasculature (Fig. 5c) was lower than that in the brain (Fig. 5b).

### Influence of Cerebral Vasculature and Material Properties of the Rat Brain

**Figure 6:**
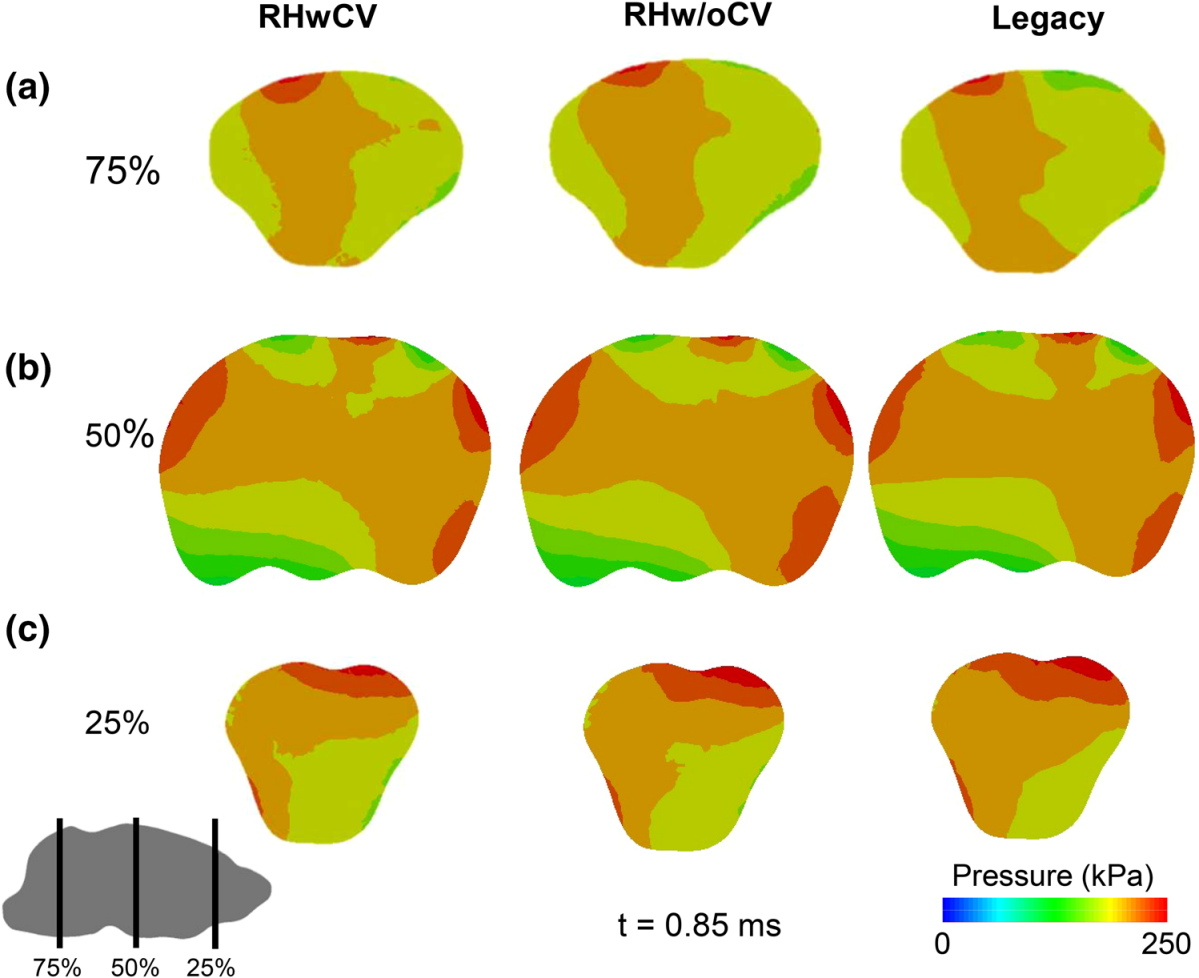
Comparison of pressures predicted by rat-head models with cerebral vasculature (RHwCV), without cerebral vasculature (RHw/oCV), and no vasculature with human material properties (legacy). Pressure predictions are for coronal planes at the (a) back, (b) center, and (c) front of the brain along the length of the anterior–posterior axis (75, 50, and 25%, respectively, from the anterior end).

**Figure 7:**
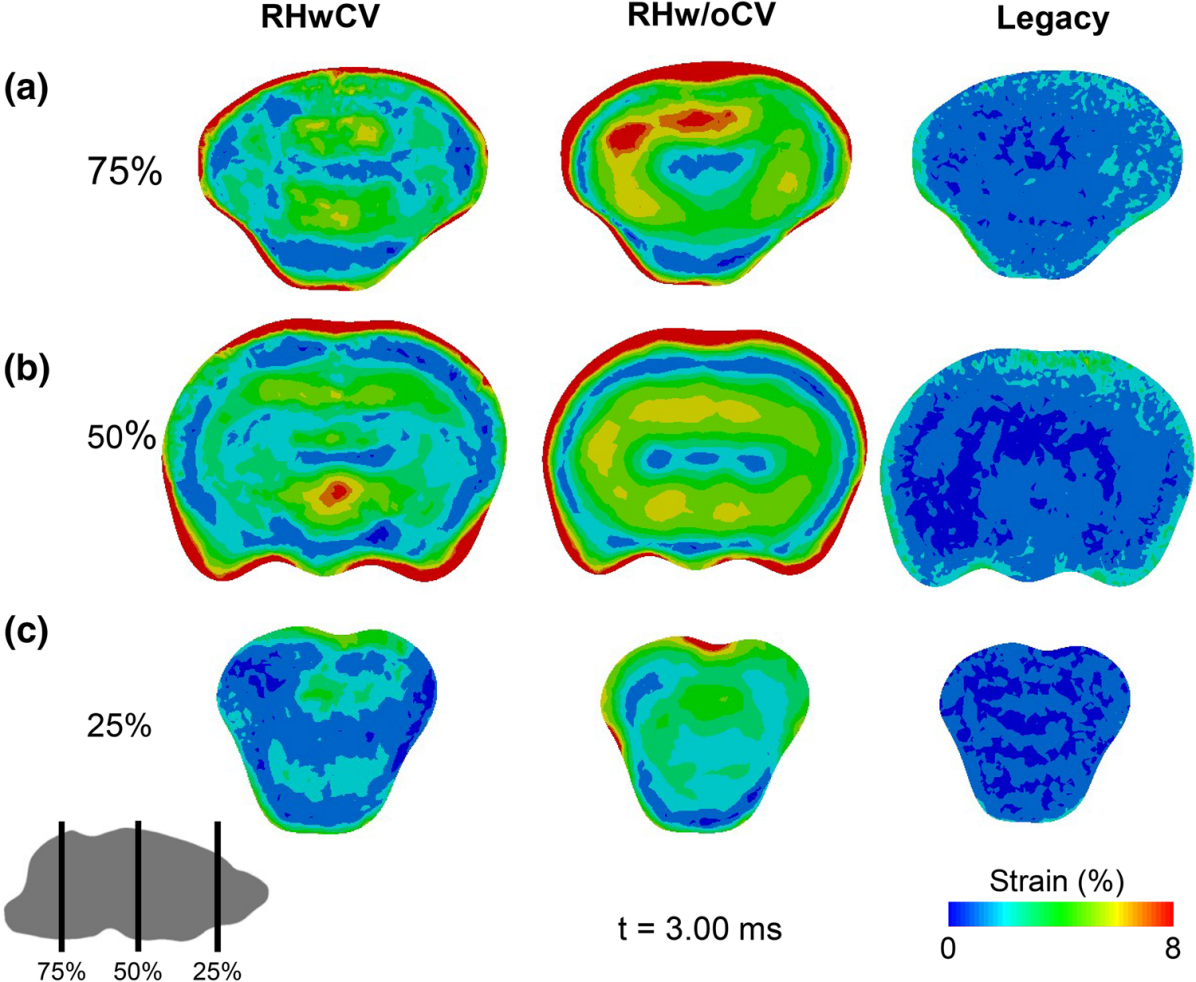
Comparison of maximum principal strain predicted by rat-head models with cerebral vasculature (RHwCV), without cerebral vasculature (RHw/oCV), and no vasculature with human material properties (legacy). Strain predictions are for coronal planes at the (a) back, (b) center, and (c) front of the brain along the length of the anterior–posterior axis (75, 50, and 25%, respectively, from the anterior end).

The cerebral vasculature and rat high-strain-rate material properties did not influence predictions of brain pressure, as is evident from the similarity in the magnitude and distribution of the pressures predicted by the RHwCV, RHw/oCV, and legacy models in coronal sections at the front, center, and back of the brain (Fig. 6). In contrast, the magnitude and distribution of the maximum principal strain were markedly influenced by the presence of cerebral vasculature and material properties (Fig. 7). The average value of the peak maximum-principal-strain differed across the three regions of the brain (Table 3). The legacy model, based on stiffer material properties of human brain tissue when compared to those of rat, predicted strains considerably lower than those of the RHwCV and RHw/oCV models.

**Table 3:** Comparison of peak maximum-principal-strain averaged over different regions of the brain for the legacy model, rat head model without cerebral vasculature (RHw/oCV), and rat head model with cerebral vasculature (RHwCV) for different vasculature thicknesses.

Model	Vasculature thickness (mm)	Average of peak maximum-principal-strain (%)		
		Cerebrum	Cerebellum	Brainstem
Legacy		1.2	2.9	2.0
RHw/oCV		6.2	6.8	6.6
RHwCV	0.010	6.1	5.7	6.3
RHwCV	0.025	5.8	5.4	6.1
RHwCV	0.050	5.5	5.1	5.8
RHwCV	0.100	5.1	4.6	5.4

**Figure 8:**
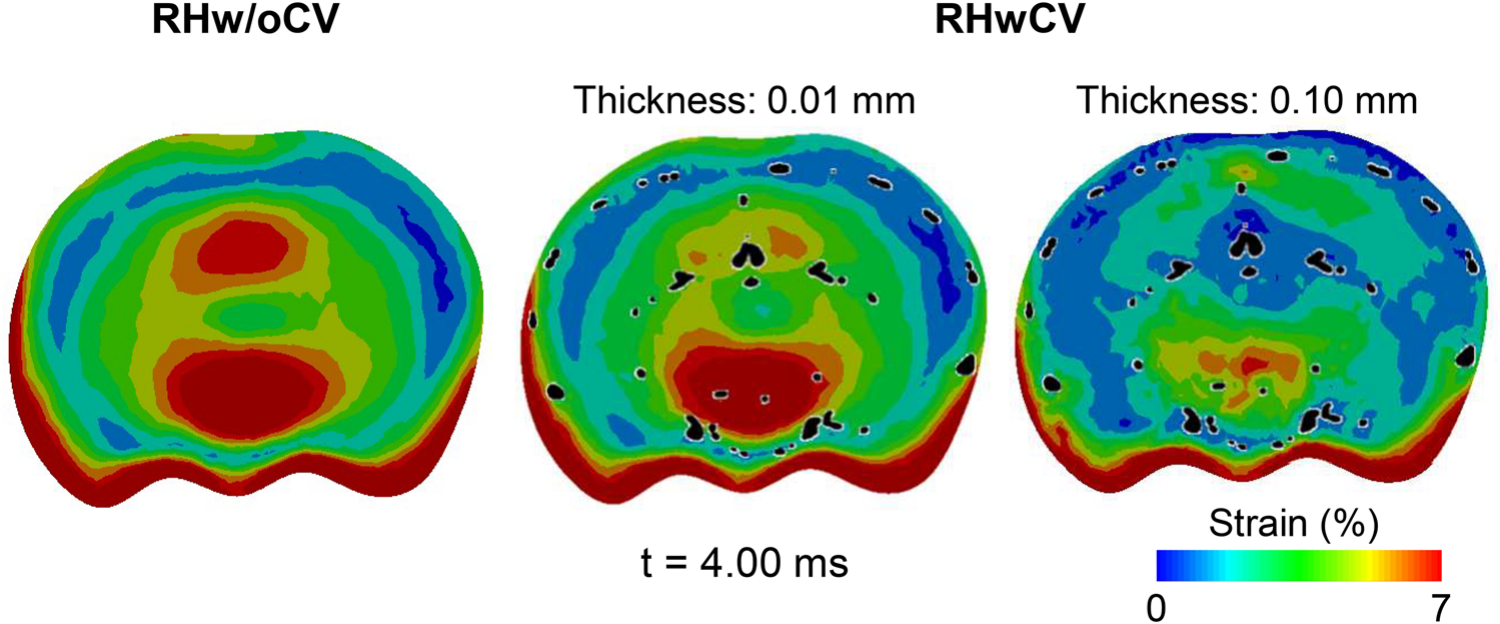
Comparison of maximum principal strain predicted by rat-head model without cerebral vasculature (RHw/oCV), and rat-head model with cerebral vasculature (RHwCV). Strain predictions are for the mid-coronal plane of the brain. The cerebral vasculature (with a thickness of 0.01 and 0.10 mm) in the RHwCV model (represented as black dots) is superimposed over the brain.

By comparing the RHwCV and RHw/oCV models, we observed that the cerebral vasculature reduced the average peak-principal-strain (Table 3). The average principal strain at all regions of the brain decreased with an increase in the thickness of the cerebral vasculature. In addition, the cerebral vasculature also influenced the spatial distribution of the strains in the brain tissue (Figs. 7 and 8). In particular, the cerebral vasculature, even with a thickness of 0.01 mm, reduced the concentration of the principal strain in the mid-coronal plane of the brain (Fig. 8). As expected, the reductions in the strains were more apparent in the brain elements with embedded vasculature (represented as black spots in Fig. 8) when compared to those elements without the vasculature. For the RHwCV model, the average peak-strain-rates for the cerebrum, cerebellum, and brainstem regions of the brain were 117, 110, and 120 s^−1^, respectively. The average model-predicted peak strain-rate for the cerebral vasculature was 30 s^−1^.

## Discussion

In this study, we developed a high-fidelity 3-D FE model of the head of a rat to capture the biomechanical responses immediately after blast-wave exposure (1–4 ms) in a shock tube. We considered four distinct regions for the rat head: the face (including the scalp and facial muscles), skull (including the facial bones), brain, and cerebral vasculature derived from µCT images. We obtained the material properties of the face and skull from the literature, consistent with previous blast simulations of the rat head.^19^ We derived the material properties of the brain and cerebral vasculature from recent high-strain-rate studies of these tissues for male Sprague–Dawley rats.^1^,^8^ To account for regional variations in the material properties of the rat brain, we divided the brain into three regions: the cerebrum, cerebellum, and brainstem. Using the FE model, we characterized the biomechanical responses of the rat brain by computing pressures, von Mises stresses, and strains, when exposed to a blast wave. We validated the FE model by comparing the simulated and experimental brain pressure–time profiles for an incident BOP of 200 kPa (Fig. 3b) and peak brain pressures for incident BOPs ranging from 100 to 230 kPa (Fig. 3c).

The rise in brain pressure predicted by the RHwCV model was instantaneous (Fig. 3b), with the pressure wave traveling from the front to the back of the brain along the direction of blast wave propagation in the shock tube. Similar to previous studies, we observed oscillations in brain pressure, which could indicate complex wave interactions in the brain, and the pressure–time profile in the rat brain closely followed the incident pressure–time profile.^17^ However, we did not observe any negative pressure in the hindbrain region of the rat brain (Fig. 4a), in contrast to results by Zhu *et al*.^25^ This discrepancy could be due to the lack of facial muscles of the rat head in the previous study, the difference in boundary conditions between the two models (i.e., fixed rat head in this study vs. free rat head), or both. The von Mises stress-time profile followed a trend similar to that of the maximum principal strain–time profile. The strains, which peaked after the peak BOP passed the rat head, initially developed in the peripheral regions of the brain, and moved deeper into the brain as time progressed (Fig. 5b). This response reflects the contributions of the translation and rotation of the rat head to the blast wave impact, in addition to the viscoelastic response of the rat brain.

Using three FE models (i.e., RHwCV, RHw/oCV, and legacy models), we quantified the effect of cerebral vasculature and high-strain-rate rat brain material properties on the biomechanical responses of the brain. For the three models, we used identical FE meshes and loading conditions, with a constant bulk modulus of 2.0 GPa for the brain. Our results showed that neither the vasculature nor the material properties of the rat brain influenced the prediction of brain pressure (Fig. 6). The similarity of the predictions indicates that brain pressure is dependent on the bulk modulus of the brain, and that the shear properties of the brain contribute little to the pressure predictions. In contrast, the maximum principal strains predicted by the three models were distinctly different from one another (Fig. 7). Our results are in agreement with prior studies that evaluated the contribution of cerebral vasculature on brain biomechanics in humans.^10^,^24^ Hua *et al*., using a surrogate human head model with an embedded network of cerebral vasculature, showed that cerebral vasculature did not influence the brain pressure but significantly altered the strain predictions during a blast-wave exposure.^10^ Furthermore, mesh-dependency and parametric studies on vessel diameter and density showed that the biomechanical predictions obtained using the surrogate human-head model were due to the presence of cerebral vasculature and not due to numerical artifacts. A comparison of the RHw/oCV and legacy models showed that the stiffer human brain tissue was responsible for a threefold reduction in the strain for the legacy model relative to the RHw/oCV model (Fig. 7). Together, these results show that cerebral vasculature and species-specific brain material properties influence the shear response of the brain of a rat exposed to a BOP.

Our analyses showed that cerebral vasculature decreased the peak maximum-principal-strain in the brain tissue when the head was exposed to a BOP (Table 3 and Fig. 7). The average strains in the cerebrum, cerebellum, and brainstem predicted by the RHwCV model were lower than those predicted by the RHw/oCV model by as much as 33%. This reduction in the strain was caused by a roughly 50-fold increase in the overall stiffness of the brain due to the cerebral vasculature (Table 1). Similar observations were also reported by other studies that evaluated the effect of cerebral vasculature on the biomechanical response of brain tissues to impact and blast loadings.^9^,^10^,^24^ For instance, the maximum principal strain predicted by a two-dimensional human head model with cerebral vasculature was lower than the strain predicted by the same model without the cerebral vasculature at the cortex, corpus callosum, and brainstem regions when the head was exposed to linear and rotational impulses.^24^

In addition to the reduction in the strain, our study showed that the cerebral vasculature considerably influenced the distribution of maximum principal strain in the brain. In the mid-coronal plane, we observed a reduction in the strain predictions for brain tissues with embedded vasculature (indicated by the black spots in Fig. 8) when compared to brain tissues without the vasculature. However, the maximum principal strain at the inferior region (i.e., bottom) of the brain was nearly identical for the RHw/oCV and RHwCV models, possibly because of the lack of cerebral vasculature in this region. These results are in agreement with the study performed by Hua *et al*., in which the difference in strain between models with and without the vasculature were considerable at regions of dense vasculature and minimal at regions of sparse vasculature.^10^ The same study also showed the importance of the orientation of the vasculature in the brain responses due to blast exposure. To summarize, the results from our study, along with the above observations, further emphasize the need to consider the cerebral vasculature, specifically an anatomically accurate 3-D network, to determine the strain responses of the rat brain exposed to blast waves.

Our simulations suffer from the following limitations. First, we did not capture cerebral vasculature with a diameter of less than 35 *µ*m (owing to the limitations of µCT imaging), assigned a uniform thickness to the shell elements of the cerebral vasculature, and did not differentiate between cerebral arteries and veins. In addition, we did not model the blood within the vessels. Second, the FE model of the rat head did not include the body, as in previous studies.^19^,^25^ Such an assumption might influence the deformation of the rat head and, in turn, the strain predictions. However, we believe that the comparative results from our simulations, which show the importance of cerebral vasculature and species-specific high-strain-rate material properties on the strain predictions, will remain valid in the absence of such an assumption. Third, due to the complex geometry of the cerebral vasculature, we coupled the vasculature and brain tissues using the embedded technique (i.e., we used an approximate approach) rather than treating the vasculature as an inclusion in the brain tissues.^5^ This limits our ability to capture the localization of stress and strain in the brain tissue that immediately surrounds the vasculature. Moreover, the embedded technique increases mass and, thereby, the stiffness of the FE model due to volume redundancy.^7^ While we cannot determine the increase in stiffness of the brain due to the added mass, we believe that the stiffening response observed for the RHwCV model (Table 3 and Fig. 8) stems from the stiffer cerebral vasculature (Table 1). Finally, we validated our FE model predictions only for brain pressure and not for strains, owing to the lack of experimental data of brain strain in response to BOPs.

To conclude, we developed a high-fidelity FE model of a rat head and characterized the biomechanical responses of the brain of the rat to blast-wave exposures. In the FE model, we explicitly represented a 3-D network of cerebral vasculature and used the high-strain-rate material properties of the rat cerebral vasculature and the rat brain tissues. Our study showed that cerebral vasculature and species-specific material properties influence the shear response, but not the pressure response in the brain. The high-fidelity FE model developed in this work will be helpful in identifying correlates between the predicted biomechanical responses and observed injuries in the rat brain and, thereby, the potential mechanisms of blast-induced TBI.

## Electronic supplementary material

Below is the link to the electronic supplementary material.

Supplementary material 1 Supplementary Fig. 1. Comparison of model-predicted and experimental stress–strain curves for (a) cerebrum, (b) cerebellum, and (c) brainstem at multiple maximum strain rates (TIFF 856 kb)

Supplementary material 2 Supplementary Fig. 2. Comparison of model-predicted and experimentally derived axial stress-stretch curves for cerebral vasculature at a maximum strain rate of 500 s^−1^ (TIFF 226 kb)
