# Supplementary figures and images for: A 3-D Rat Brain Model for Blast-Wave Exposure: Effects of Brain Vasculature and Material Properties
Source: Ann Biomed Eng. 2019 May 3;47(9):2033–44. doi: 10.1007/s10439-019-02277-2 (PMC6757019; doi:10.1007/s10439-019-02277-2)

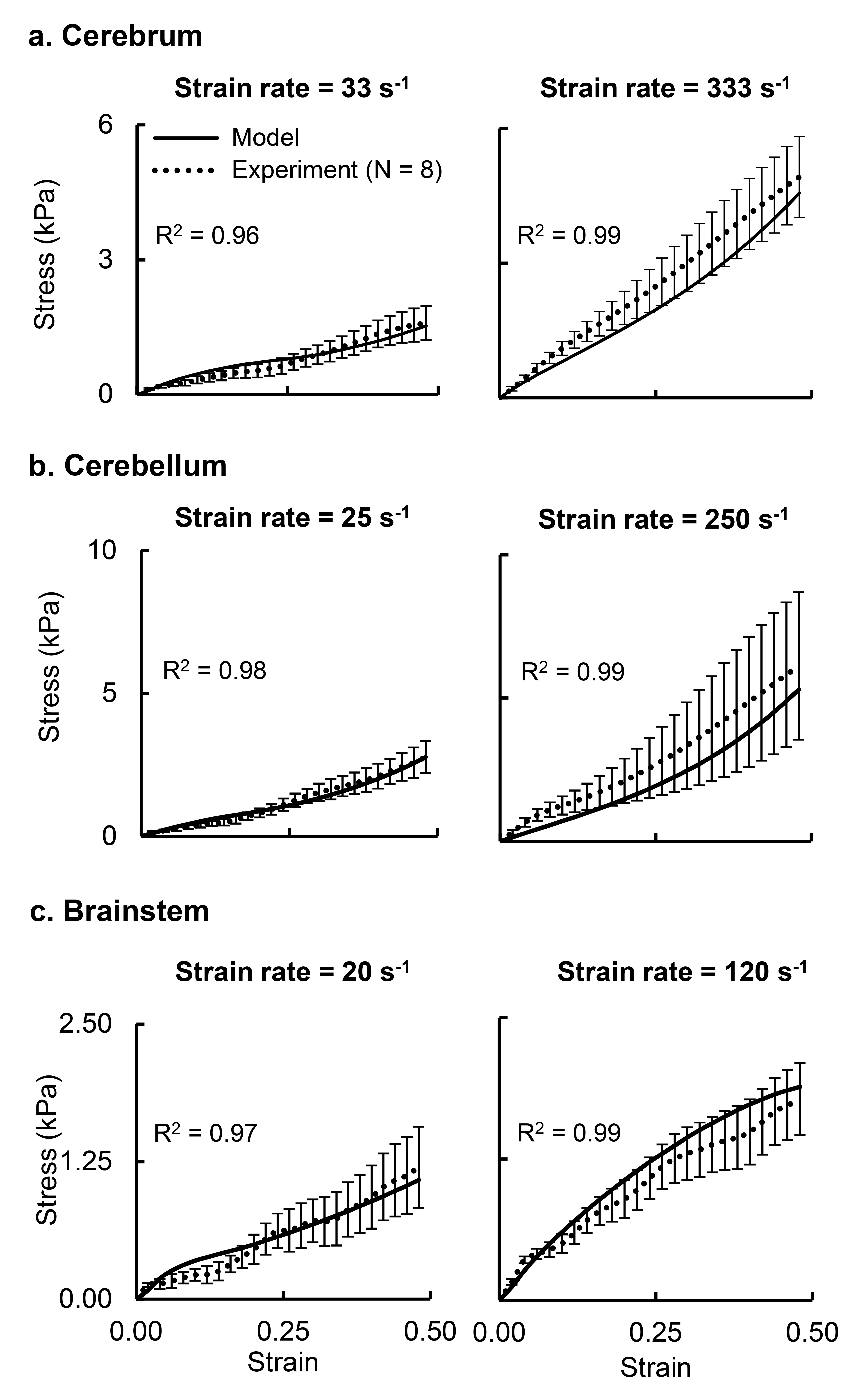

Supplement: Supplementary file 1 — Supplementary material 1 Supplementary Fig. 1. Comparison of model-predicted and experimental stress–strain curves for (a) cerebrum, (b) cerebellum, and (c) brainstem at multiple maximum strain rates (TIFF 856 kb) [file 10439_2019_2277_MOESM1_ESM.tif]

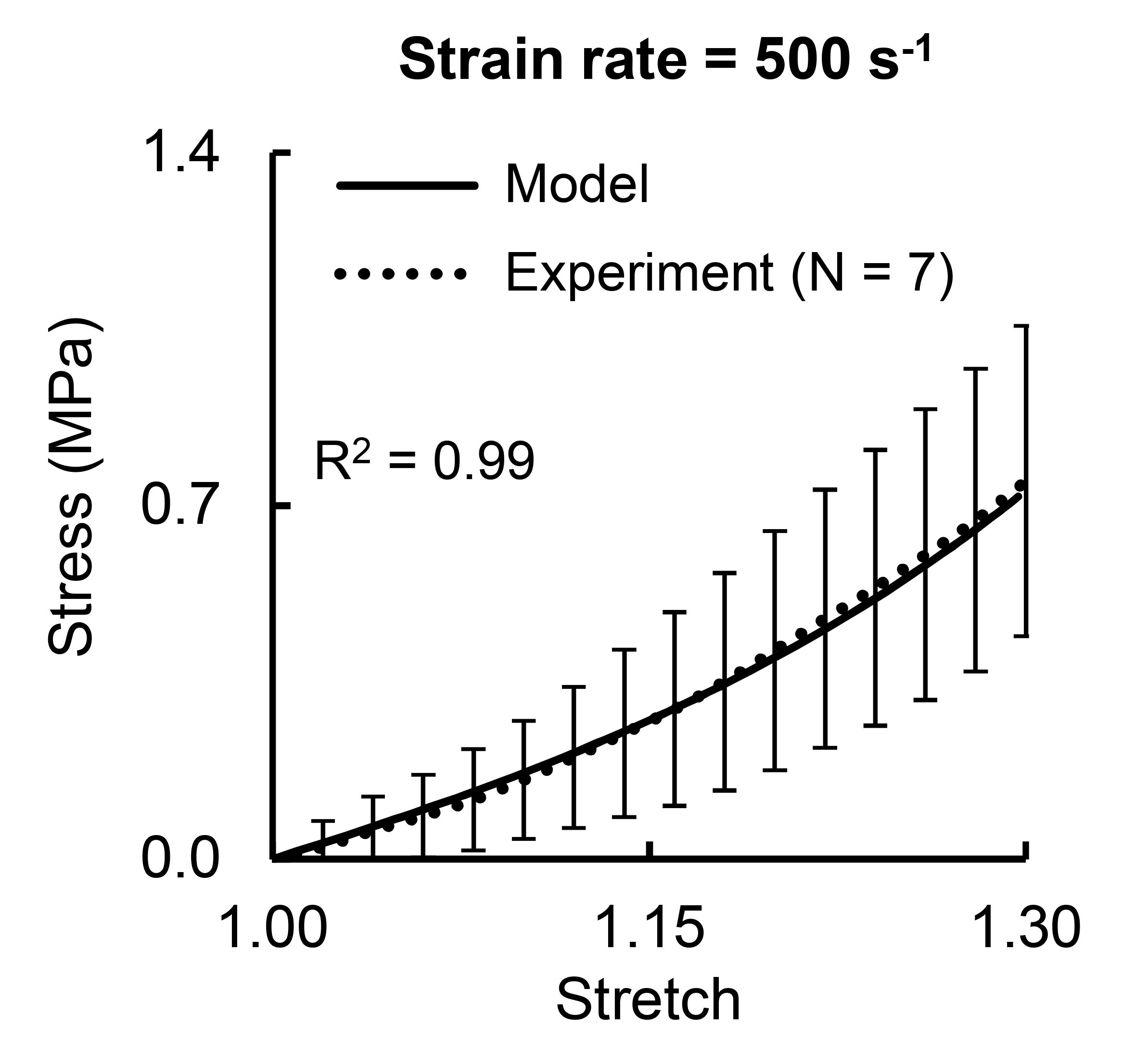

Supplement: Supplementary file 2 — Supplementary material 2 Supplementary Fig. 2. Comparison of model-predicted and experimentally derived axial stress-stretch curves for cerebral vasculature at a maximum strain rate of 500 s−1 (TIFF 226 kb) [file 10439_2019_2277_MOESM2_ESM.tif]
